# A Future Picture: A Review of Current Generative Adversarial Neural Networks in Vitreoretinal Pathologies and Their Future Potentials

**DOI:** 10.3390/biomedicines13020284

**Published:** 2025-01-24

**Authors:** Raheem Remtulla, Adam Samet, Merve Kulbay, Arjin Akdag, Adam Hocini, Anton Volniansky, Shigufa Kahn Ali, Cynthia X. Qian

**Affiliations:** 1 Department of Ophthalmology & Visual Sciences, McGill University, Montreal, QC H4A 3SE, Canada; raheem.remtulla@mail.mcgill.ca (R.R.); merve.kulbay@mail.mcgill.ca (M.K.); 2 Centre de Recherche de l’Hôpital Maisonneuve-Rosemont, Université de Montréal, Montreal, QC H1T 2M4, Canada; 3 Faculty of Medicine and Health Sciences, McGill University, Montreal, QC H3G 2M1, Canada; 4 Faculty of Medicine, Université de Montréal, Montreal, QC H3T 1J4, Canada; 5 Department of Psychiatry, Université Laval, Quebec City, QC G1V 0A6, Canada; 6 Department of Ophthalmology, Centre Universitaire d’Ophtalmologie (CUO), Hôpital Maisonneuve-Rosemont, University of Montreal, Montreal, QC H1T 2M4, Canada

**Keywords:** generative adversarial neural networks, predictive modeling, computer vision, diagnostic imaging, vitreoretinal pathology

## Abstract

Machine learning has transformed ophthalmology, particularly in predictive and discriminatory models for vitreoretinal pathologies. However, generative modeling, especially generative adversarial networks (GANs), remains underexplored. GANs consist of two neural networks—the generator and discriminator—that work in opposition to synthesize highly realistic images. These synthetic images can enhance diagnostic accuracy, expand the capabilities of imaging technologies, and predict treatment responses. GANs have already been applied to fundus imaging, optical coherence tomography (OCT), and fluorescein autofluorescence (FA). Despite their potential, GANs face challenges in reliability and accuracy. This review explores GAN architecture, their advantages over other deep learning models, and their clinical applications in retinal disease diagnosis and treatment monitoring. Furthermore, we discuss the limitations of current GAN models and propose novel applications combining GANs with OCT, OCT-angiography, fluorescein angiography, fundus imaging, electroretinograms, visual fields, and indocyanine green angiography.

## 1. Introduction

The potential applications of artificial intelligence (AI) in medicine have significantly increased in recent years. Novel AI-derived models have been developed with increasing computational power over the years. Advances in image classification (e.g., improvement of top-five accuracy on ImageNet from ~84% (AlexNet, 2012) to over 99% (EfficientNet, 2022)) and reinforcement learning (e.g., the surge in Atari games performance from ~200% of the human score (DQN, 2015) to ~800% of the human score (2023)) have been reported [1,2]. Initially, the academic interest was directed towards discriminatory models, which allow for discrimination between variables and perform classifications. These discriminatory models include deep convolutional neural networks (DCNNs) [3], convolutional neural networks (CNNs) [4,5], artificial neural networks (ANNs) [6,7], random forest [8], and decision tree [9]. Several of these models have already been integrated into clinical practice within the field of ophthalmology [10,11,12]. In 2018, the Food and Drug administration (FDA) approved for clinical practice the first-ever neural network, an algorithm capable of early identification of diabetic retinopathy (DR) with a sensitivity ranging from 92% to 93% and specificity ranging from 89% to 94% [13]. The incorporation of AI in health care also occurred in other countries, such as India, where similar networks allow screening for the early identification and treatment of patients with DR [13,14,15]. Further machine learning models have been used in screening patients with retinopathy of prematurity (ROP) [16], age-related macular degeneration (AMD) [17], and vein occlusions [18,19,20]. The extent of machine learning algorithms is not restricted to diagnosis; algorithms can also perform predictions regarding therapy response. Indeed, machine learning approaches have demonstrated the ability to predict response to anti-vascular endothelial growth factor (VEGF) in patients with AMD [21,22,23,24].

**Figure 1:**
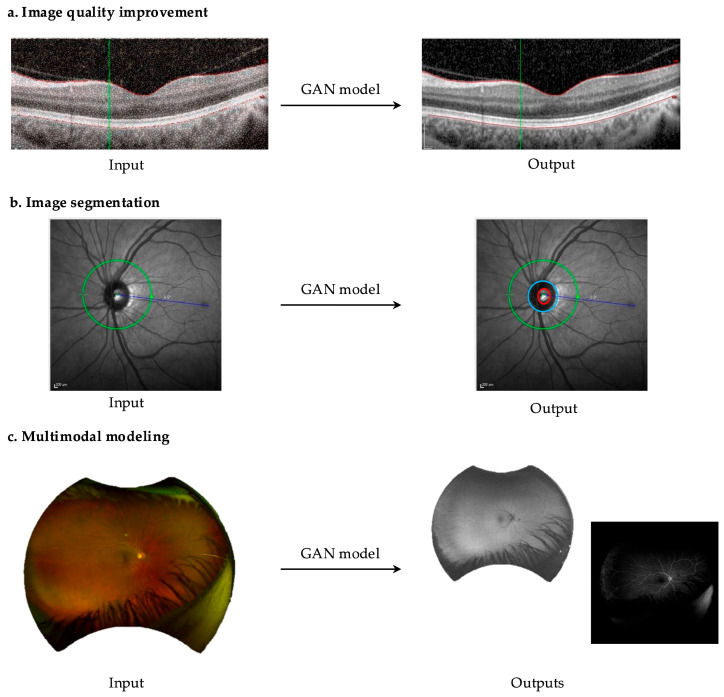
Applications of Generative Adversarial Networks in Ophthalmology. GANs can enhance image quality by reducing noise and artifacts (**a**), perform image segmentation for structure identification, such as cup (red) and disc (blue) assessment when assessing an optic nerve (nerve) (**b**), and generate imaging modalities from other inputs (**c**), supporting clinical diagnosis, disease management, and physician training.

In recent years, generative models have also gained worldwide interest. Generative models learn patterns from a dataset and generate new but similar data [25,26,27,28,29,30]. Despite the large interest in the generative data science field, such as building models with text and language inputs for generative models [31], image generation has not gathered as much attention in the field of medicine compared to conventional discriminatory models. Image generation has a multitude of clinical applications, particularly in image-based specialties such as radiology and ophthalmology [32,33]. Generative adversarial networks (GANs) are the most common models used to generate realistic synthetic images. These models have proven their high degree of realism when generating high-resolution images [34,35]. In radiology, GANs have been demonstrated to improve the quality of diagnostic imaging by reducing noise and artifacts [36,37,38]. They have even been used to convert images between computational tomography (CT) and magnetic resonance imaging (MRI) acquisitions [39]. In ophthalmology, GANs have been implicated in image segmentation to identify structures such as optic nerve cupping [40], retinal vessels [41], and the meibomian glands [42]. GANs have also demonstrated the ability to generate fluorescein angiograms from fundus photos [43], as well as predicting response to treatments such as anti-VEGF therapy. Given that the applications of GANs in ophthalmology are likely to expand with increasing databases and computing power (Figure 1), it is essential to provide an overview that explains how these networks function and addresses their common pitfalls within the context of ophthalmology. The aim of this review is to serve as a primer for vitreoretinal surgeons by focusing on three key objectives: (1) to provide a foundational understanding of GAN architecture and functionality, (2) to explore clinical applications in retinal imaging, and (3) to highlight future directions and limitations of GANs in ophthalmology.

## 2. Fundamental Concepts on Generative Adversarial Networks

### 2.1. Basic Terminology of GANs and Neural Networks

To help navigate through the technical sections involving GANs, Table 1 summarizes common terminology encountered. Most terms will also be defined individually within the text when presented for the first time. We suggest referring to the references provided in Table 1 for more in-depth explanations of GAN architecture.

### 2.2. How Do GANs Work? Explanation with the Counterfeit’s Analogy

**Figure 2:**
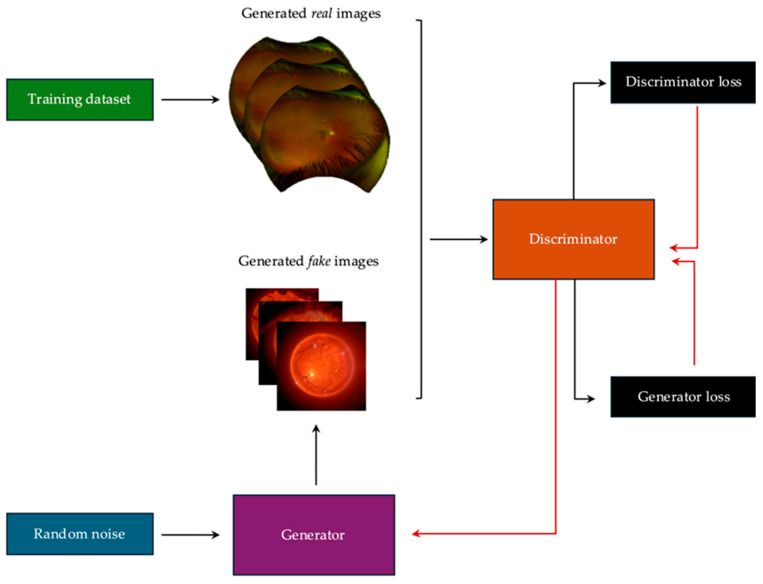
Schematic representation of a generative adversarial network architecture applied to ophthalmology. GANs consist of two primary components: a generator and a discriminator. The generator produces synthetic images starting from random noise and aims to generate outputs that resemble real images. Real images are sourced from the training dataset, while the discriminator is tasked with distinguishing between real images and synthetic images created by the generator. The discriminator evaluates each input image and calculates the likelihood of it being real or fake. Feedback, or backpropagation (indicated by red arrows), flows from the discriminator to the generator, allowing the generator to improve its image synthesis over successive iterations. The goal is for the generator to produce images that are indistinguishable from real ones. The fake images shown here were generated using a Stable Diffusion model (accessible at https://app.aitubo.ai/create/ accessed on 10 October 2024). The schematic captures the interplay between the generator and discriminator in a GAN setup, particularly in the context of ophthalmic imaging. The Figure was created based on the cited reference [36].

GANs were developed in 2014 in Montreal (Quebec, Canada) by Ian Goodfellow, a PhD student, under the supervision of Yoshua Bengio and his team [53]. GANs function on an adversarial game theory model between two CNNs: a generator and a discriminator (Figure 2) [33,36,53,54,55,56,57]. The analogy often employed to illustrate this concept involves a counterfeit representing the generator and a police officer representing the discriminator. The purpose of the counterfeit is to produce an authentic copy of an object, while the purpose of the police officer is to distinguish the replica from the original. As the counterfeit begins to learn his art, his first attempts are easily distinguishable from the original objects, and the police officer identifies the replica. However, the feedback from the police officer about the counterfeit serves as a quality control step and forces the counterfeiter to improve his replica. Thus, after several attempts, the counterfeit gains experience and eventually masters his art. The competition between these two antagonists is the origin of the term adversarial in GANs and allows the counterfeit (i.e., generator) to fulfill its function. The analogy aside, in imaging, the purpose of the generator is to generate synthetic images based on a provided input (e.g., another image, text, speech, or video files) [33,36,53,54,55,56,57]. The generator model uses a distribution of data to perform internal comparisons and provide a candidate image close to a real counterpart [33,36,53,54,55,56,57]. The purpose of a discriminator is to differentiate between a real and a synthetic image by calculating the probability that the provided image is real [33,36,53,54,55,56,57]. Feedback from the discriminator to the generator allows model improvement with each classification iteration. Mathematically, the generator can be defined as a function *G*(*z*; *θ*_*g*_), where z is the input noise vector and *θ*_*g*_ represents the model parameters [58,59,60]. Similarly, the discriminator is a function *D*(*x*; *θ*_*d*_), where *x* represents the input image and *θ*_*d*_ represents the discriminator parameters. The optimization goal for GANs is a minimax game defined as:

$$\frac{min}{G}\frac{max}{D}E_{x}{\sim P}_{data}\left[\log D\left(x\right)\right]+E_{z}\sim P_{z}\left[\log\left(1-D\left(G\left(z\right)\right)\right)\right]$$

Here, *P*_data_ represents the distribution of real images, whereas *P*_*z*_ is the prior distribution of the noise vector. The generator aims to minimize the loss while the discriminator maximizes it [33,36,53,54,55,56,57]. Improvement of the generator is usually performed by altering the weights in the nodes of the hidden layers of the model based on provided feedback [33,36,53,54,55,56,57]. This backward adjustment of the weights of the model generator is called backpropagation [33,36,53,54,55,56,57]. The mathematic optimization technique used for weight update is the partial derivatives of the loss function to each weight, which is called gradient descent [33,36,53,54,55,56,57]. The loss function is the function that evaluates the algorithm’s performance when comparing its output to the ground truth [33,36,53,54,55,56,57]. For ophthalmologic applications, the loss function can take various forms, such as pixel-wise mean squared error (MSE) for reconstruction tasks or perceptual loss to enhance the realism of generated OCT images. Specific loss functions tailored to clinical applications, like the Structural Similarity Index (SSIM) loss, have also been employed for improved clinical interpretability [33,36,53,54,55,56,57]. Typical GANs are based on generator and discriminator CNNs; however, DCNNs can also be employed [33,36,53,54,55,56,57]. CNNs are used for image classification and feature extraction, while DCNNs are a deeper version of CNNs with more layers, enabling them to learn more complex and abstract features for tasks like image generation [33,36,53,54,55,56,57].

Translation of GANs to ophthalmology can take various forms. For example, in the case of a GAN built to predict the macular OCT findings in a patient with DR from fundus photos, the role of the generator is to compare provided fundus photos (inputs) to the available training set and learn to generate a new, realistic, and corresponding OCT image [33,36,53,54,55,56,57]. The role of the discriminator is to provide feedback to the generator during training regarding the appropriateness of the generated OCT images (i.e., look realistic) and accuracy (i.e., if they correspond to appropriate fundus photos) [33,36,53,54,55,56,57].

## 3. Generative Adversarial Network Model Training

GAN training encompasses two main steps: discriminator training and generator training [33,36,53,54,55,56,57]. Usually, GAN generators and discriminators are trained separately, but simultaneous is also possible. This is known as alternate training [33,36,53,54,55,56,57]. In this section, we will delve into the fundamental principles underlying each process.

### 3.1. Discriminator Training

Training a discriminator is the first step in training GANs. The generator model functionality is kept constant while the functionality of the discriminator is allowed to fluctuate to create an “ideal” discriminator [33,36,53,54,55,56,57]. Input data for discriminator training comes from both real examples (i.e., inputs to classify as positive examples) and from the generator (i.e., inputs to classify as negative examples) [33,36,53,54,55,56,57]. Both data are then classified by the discriminator; their predictions are compared to the ground truth to establish total loss [33,36,53,54,55,56,57]. Through backpropagation, the discriminator is penalized for errors to which the weights in decision-making are altered [33,36,53,54,55,56,57]. Each cycle during which the complete training dataset is fed to the discriminator for training is called an epoch [33,36,53,54,55,56,57]. Through the training process, multiple epochs occur, and with each cycle, the performance of the discriminator improves [33,36,53,54,55,56,57]. Training stops when additional epochs add little value to performance or when the maximum allowed epochs is reached [33,36,53,54,55,56,57]. Batch size, a critical parameter determining the number of samples processed at a time, significantly affects the model’s performance and training stability. Optimizing batch size is non-trivial and requires empirical testing to balance computational efficiency with model accuracy. A computer scientist is best equipped to fine-tune this parameter [33,36,53,54,55,56,57]. In summary, discriminator training involves iterative optimization using real and generated data to improve its ability to classify inputs accurately, with performance stabilizing after sufficient epochs.

### 3.2. Generator Training

In the training process, the generative model is provided with an input and attempts to produce a plausible image output [33,36,53,54,55,56,57]. As mentioned previously, this output acts as a training example for the discriminator model, which then attempts to distinguish the generator’s synthetic output from a real image [33,36,53,54,55,56,57]. The discriminator penalizes the generator for being recognized [33,36,53,54,55,56,57]. A similar process for the discriminator happens with the generator: weight adjustments through backpropagation allow improvement of the generator, and training stops when each additional epoch adds little value to the performance outcome or when the maximum allowed epochs are reached [33,36,53,54,55,56,57]. Changes to the discriminator through training would make reaching an appropriate endpoint difficult for the generator. Therefore, the discriminator model functionality is kept constant while the generator functionality is altered [33,36,53,54,55,56,57]. In summary, generator training focuses on improving the model’s ability to produce realistic outputs by minimizing loss through iterative updates, aiming for stable convergence (i.e., the point where the loss function is minimized and training parameters reach a stable state, allowing accurate generations).

### 3.3. Alternate Training

The alternative training approach is now a standard of practice for modern GANs and consists of training the generator and discriminator simultaneously [33,57,61]. In this process, the discriminator may be trained for a set number of epochs, and then the same process is undertaken to train the generator [33,57,61]. This process is repeated a multitude of times until there is minimal improvement in the functionality of the generator [33,57,61]. The advantage of this method is that it theoretically improves the functionality of the end point generator, as both the discriminator and generator are faced with more challenging tasks as training progresses [33,57,61]. However, this can make convergence more challenging [33,57,61]. Learning rate selection, another key parameter, directly influences the convergence speed and stability of training [33,57,61]. A high learning rate may cause instability, while a low rate could lead to slow convergence [33,57,61]. Learning rate schedules or adaptive methods, such as Adam optimizers, are preferred for GAN training. Determining the optimal configuration often requires expertise in machine learning [33,57,61]. As the performance of the discriminator decreases, the generator function improves [33,57,61]. Through the training process, the generator function continues to improve by learning from feedback provided by the discriminator [33,57,61]. As a result, the generator can produce images that closely mimic real images, making them nearly indiscernible to the discriminator and, potentially, to human observers [33,57,61]. At this point, even the most ideal discriminator performance would be no better than chance or 50% [33,57,61]. If the network continues to train, then the generator model will adapt to feedback from the discriminator, which will provide inaccurate feedback [33,57,61]. This phenomenon decreases the overall functionality of the generator model and illustrates an example of failure to converge [33,57,61]. While this method enhances both models, it can complicate convergence due to the adversarial dynamics. Variants of gradient descent, such as RMSProp or AdaGrad, are often employed to stabilize training. The choice of optimization algorithm is highly dependent on the application and data characteristics. This task is best left to experts in machine learning [33,57,61]. In summary, alternate training of GANs improves both models’ performance through simultaneous optimization, but adversarial dynamics can complicate convergence.

### 3.4. Categories of Input

Inputs serve as a starting point for the generator to produce synthetic images [62]. In the first models, the inputs of GANs were noise images [62]. Through training, the GANs learn to develop a coherent image from that noise [62]. In the field of ophthalmology, such GANs have limited value since noise base inputs are unlikely to provide valuable clinical value. More practical applications of GAN inputs typically include text, image, or video files. During training, these data inputs are paired with target image outputs, with the goal of the GAN being to generate the target image [62]. As with all machine learning programs, there is a significant reliance on the underlying data.

## 4. Evaluating GANs

### 4.1. Evaluative Measures

Two terms have been used to describe the “realness” of outputs from GANs: fidelity and diversity [33,56,57,63]. Fidelity often refers to the overall quality of the produced image [33,56,57,63]. This can depend on a variety of features, such as, but not limited to, texture, structure, and detail [56,62,64]. Diversity refers to the variety of images that GANs can produce. GANs that produce only one type of image, or duplicates of a similar image, have limited practical value and thus are considered to have low diversity [56,62,64]. Functional GANs must be able to produce images with both great fidelity and diversity. In machine learning discriminatory problems, total accuracy, sensitivity, and specificity are commonly used to measure the performance of the neural network [56,62,64]. In generative neural networks, the evaluation task is more challenging [56,62,64]. The fundamental issue is that a “real image” is difficult to define [56,62,64]. Therefore, unlike classification tasks, where an algorithm can clearly distinguish between two categories, evaluating whether an image is ’real’ or ’not real’ involves subjective judgment (i.e., human input) and assessment of multiple complex characteristics of the image [56,62,64].

### 4.2. Qualitative vs. Quantitative Methods (Pixel-Wise Loss)

There are two categories of methods for evaluating GANs: qualitative methods and quantitative methods [56,62,64]. Qualitative means usually include human raters and encompass, but are not limited to, methods that use masked graders to evaluate outputs from GANs [56,62,64]. It is common practice to compare outputs from GANs to their ground truth images. However, such methods are human labor intensive and are subject to bias [56,62,64]. Conversely, quantitative methods do not involve human judgment but can be difficult to implement [56,62,64]. Quantitative methods encompass tools that can compare pixel variations of GAN outputs to their corresponding ground truth counterparts, which needs to be available for comparison [56,62,64]. This pixel-by-pixel evaluation of deviation is called pixel-wise loss. Pixel-wise loss allows for a more objective evaluation of a GAN’s performance. However, this approach is not a flawless metric of GAN performance since simple deviations in pixel values from the ground truth do not directly correlate to realism [33,56,57,63]. A synthetic image may have limited pixel variation from its ground truth counterpart, but incoherency in a small area can question the realism of the entire image [33,56,57,63]. For example, if a GAN generates an image of a human hand with six fingers instead of five, the realism of the entire picture would be questioned. Similarly, if a synthetic image has a large pixel variation but no incoherence in it, the pixel-wise loss will underestimate the realism [33,56,57,63].

### 4.3. Other Objective Methods

Other objective measures include using an SSIM, the Inception Score (IS), or the Frechet Inception Distance (FID). SSI compares GAN outputs to their ground truth counter parts by looking at features such as luminance, contrast, and structure between the two images [65]. Although this does allow for a more reliable metric than simple pixel-to-pixel variation, this method still does not measure the realness of the image [65]. The IS uses a classification model pre-trained on a large annotated dataset of images (e.g., ImageNET for Inception v3) to evaluate the generated outputs [66,67,68]. The Inception v3 model has become popular since this approach has been highly correlated with human evaluators [66,67,68]. However, an important limitation of the clinical translation of Inception V3 is the highly dependent nature of the model on the database used in pretraining [66,67,68]. Since ImageNET has limited medical images, the model is, unfortunately, sometimes of limited value in medical applications [66,67,68].

FID has also become a popular outcome measure for GANs [33,69]. In this methodology, an Inception v3 model is employed to extract features from both the real and generated images and subsequently generate a distribution [33,69]. The difference between these distributions is known as the FID [33,69]. One advantage of FID is that it can identify mode collapse, unlike IS [33,69].

Overfitting refers to an undesirable behavior of an algorithm that fails to generalize and fits too closely to its training data, resulting in it being unable to correctly perform accurate prediction [57,62,63,64,70]. A helpful analogy to understand the concept is an engineering student who memorized all the physics exercises in his chapter instead of understanding the physics itself. Currently, there is no simple measure to evaluate overfitting in GANs, and all metrics have their own core flaws and no clear gold standard [57,62,63,64,70]. The ideal metric for evaluating the functionality of a GAN should be determined by a multitude of factors, including the network architecture, the nature of the presented data, and the task performed [57,62,63,64,70]. Unfortunately, specific threshold values for retinal imaging cannot be universally standardized due to the inherent variability in imaging modalities and the diverse range of disease entities encountered in clinical practice. Each imaging technique has its own set of parameters and sensitivities that influence threshold determination. Moreover, the threshold values often need to be tailored to the specific pathology under investigation, as different retinal diseases may require distinct diagnostic criteria. Given the complexity of optimizing these thresholds across various modalities and diseases, input from an expert in computer science is essential to develop and fine-tune algorithms that can dynamically adjust and apply these parameters for improved diagnostic precision.

## 5. Loss Function

Loss functions evaluate models (generator or discriminator) through training [33,56,57,63]. The loss functions not only allow the user to evaluate the performance of a generator but also provide the necessary feedback to the generator model for optimization through backpropagation and gradient descent [33,56,57,63]. These loss functions represent the difference between the distribution of produced data and the distribution of real data [33,56,57,63]. The objective of the training is to build a discriminator whose performance is maximized and who is unable to distinguish between real vs fake data provided by the generator and whose performance is also maximized [33,56,57,63]. There are three primary loss functions that have been employed that we will discuss in the ensuing sections [33,56,57,63].

### 5.1. Minimax Loss

The first loss function that was described in the paper that introduced GANs by Ian Goodfellow is called minimax loss—we suggest referring to Section 2.1 for its mathematical explanation [53,71]. However, this loss function had a flaw: occasionally, at the beginning of the training, the discriminator performed his task of discriminating synthetic images so well that the generator failed to catch up (i.e., produce images), thus stopping prematurely to training [53,71]. To alleviate the previous issue, modified minimax loss and Wasserstein loss functions were developed [53,71].

### 5.2. Modified Minimax Loss and Wasserstein Loss

Modified minimax loss attempts to counteract early termination of training by determining the generator’s loss from the probability that a discriminator believes a synthetic image is real rather than comparing the direct distribution of real data points to synthetic ones [57,71,72]. Mathematically, this approach is defined as LG = −Ez∼Pz[log(D(G(z)))] [57,71,72]. Wasserstein loss, often referenced as Wasserstein GAN or wGAN since it is an extension of a classic GAN, attempts to counteract the previous issue with another approach [57,71,72]. The output of a Wasserstein GAN discriminator is different from the output of a classic GAN discriminator [57,71,72]. The first provides an output score of being “real” (higher score = more realistic image), while a classic GAN provides a probability, an odd between 0 and 1, of the image being real [57,71,72]. Mathematically, Wasserstein loss is defined as LG = −Ez∼Pz[D(G(z))]. Wasserstein loss has been demonstrated to decrease the probability of early termination of training [57,71,72]. Thresholds can be created to define whether an image is real or synthetic. The goal of the Wasserstein GAN’s discriminator is to maximize its score accuracy [57,71,72].

### 5.3. Other Loss Functions

Other variations in loss functions include the use of mean squared loss [57]. For standard GANs, log loss is used in the log function through training [57]. However, there is evidence suggesting that employing mean squared loss increases the probability that synthetic images will approximate their ground truth counterparts [57]. All loss functions can be useful in evaluating the functionality of a GAN; however, the primary function of loss functions is to guide the training process [57].

## 6. Overview of GANs Applications in Ophthalmology

### 6.1. Image Quality Improvement

**Figure 3:**
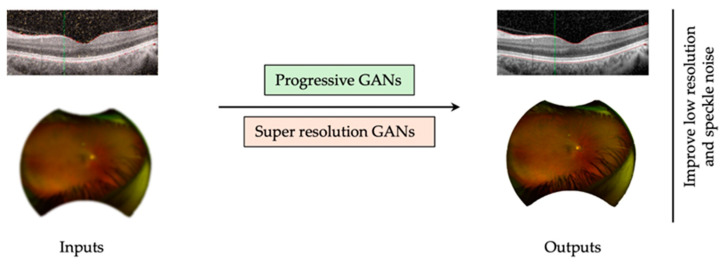
Generative adversarial networks improve imaging quality in the field of ophthalmology. Generative adversarial networks (GANs) can be applied to optical coherence tomography images and fundus photos by decreasing speckle noise induced by media opacities during acquisition, as well as removing artifacts induced during acquisition.

Super-resolution and progressive GANs are specific subtypes of GANs aimed at increasing the resolution of images. However, the structure of these GANs is more dynamic, where resolution is improved progressively through the training process [73,74]. In the field of medical imaging, these types of GANs have applications for improving image quality and decreasing artifacts [62,73,74]. In ophthalmology, image quality improvement can enhance the resolution of images (Figure 3), such as optic disc photography that can be limited by patient cooperation, small pupils, or media opacities [75]. GANs can also improve retinal images taken by a handheld fundus camera in limited imaging conditions [76]. Similarly, in OCT images with low resolution and speckle noise, due to media opacities, which can limit accurate diagnosis, super resolution GANs can output high resolution OCT images allowing better classification [77].

### 6.2. Inpainting

**Figure 4:**
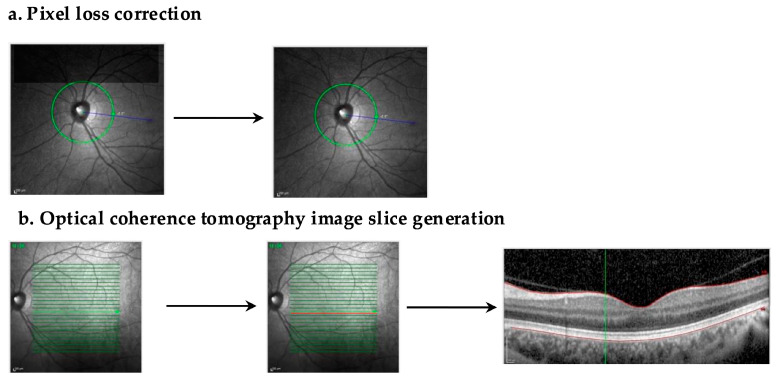
Inpainting application illustrations applied to ophthalmology. Inpainting can be used for various applications in ophthalmology, such as filling in photos with black areas (**a**) and generating intermediate synthetic slices between optical coherence tomography acquisition images (**b**).

GANs designed for inpainting are those used to fill-in images with missing pixels or artifacts (Figure 4) [78]. Furthermore, GANs can be applied to generate intermediate synthetic slices in between two imaged-derived slices [78]. This technology could be used to generate thinner slices from an OCT from a thicker imaging protocol or to generate phases of fluorescein angiography that were not captured [78].

### 6.3. Conditional GANs

cGANs function similarly to standard GANs; however, they differ in that they allow the user to define conditioning variables to alter training [79,80,81]. This means that the GAN can be trained with more information than just the data provided [79,80,81]. Theoretically, a condition can be applied to any variable in training; however, research evaluating the impact of a single status variable, images of multiple domains, masked images, and heat-map-guided images is currently being conducted [79,80,81]. The ability to provide additional information to the GAN allows for more control over the training process and the desired characteristics of the final model [79,80,81].

### 6.4. Multimodal GANs

Recent applications of GANs have taken advantage of the multimodal inputs. GANs can be used for translation between text, images, speech, and video [62,64,70]. In the medical field, speech-to-text GANs have been implicated in generating clinical notes by recording audio files from clinical interactions [82]. In the field of vision science, video-to-speech GANs have been used to help patients with visual impairment navigate their environment [83].

### 6.5. Translational GANs

Translational GANs allow for the translation of one input to another output with a defined property [84,85]. The majority of the research body for medical applications of GANs has centered around these types of GANs, with a focus on image-to-image models [84,85]. Image-to-image GANs have been used to convert ultrasound images and CT scans into MRI images [84,85]. In the field of ophthalmology, studies have demonstrated that fundus photos can be translated into corresponding fluorescein autofluorescence images [84,85]. One of the most well-studied translational GANs is CycleGAN due to its simplicity and high performance in generating plausible outputs [84,85]. CycleGAN functions to translate images from a data distribution into images that belong to another data distribution of a differing characteristic [84,85]. Unlike other GANs, CycleGAN has two generator and two discriminator functions [84,85]. In this setup, the output of the first generator is used as the input for the second generator [84,85]. For this reason, CycleGAN does not require labels or pairwise correspondence, as the cycle between the generators allows the model to learn the characteristics of the two separate image domains [84,85]. This makes producing datasets for CycleGAN simpler and allows for the development of networks for denoising without paired datasets [84,85]. For translational image-to-image GANs, loss can be evaluated by comparing the pixels of the synthetic images to their ground truth counterparts [84,85].

## 7. Current Generative Adversarial Networks Applications with Different Imaging and Functional Testing Modalities in Ophthalmology and Retina

In recent years, numerous studies have sought to develop GANs to generate various imaging results based on the available modalities in ophthalmology. Given the enormous body of literature on the topic, in this section, we outline the most recent and/or most accurate and powerful GANs being developed for potential use in clinical practice based on the various imaging modalities. However, one should be aware that GANs in ophthalmology currently being developed can be applied to various retinal pathologies, mainly AMD, DR, vascular occlusive retinal diseases, and inherited retinal diseases (IRD). The aim of this section is to provide an overview of the clinical potential of GANs in ophthalmology.

### 7.1. Fundus Autofluorescence

Fundus autofluorescence (FAF) is a non-invasive imaging technique that reduces the risk of potential complications and adverse effects when compared to the FA imaging technique. Limitations of FAF imaging include lower signal strength than FA and greater susceptibility to artifacts from anterior structures [86]. This imaging technique uses the fluorescent proprieties of lipofuscin in the retinal pigment epithelium (RPE) to create an image. The image abnormalities found in FAF can be further subdivided into two groups based on their appearance in autofluorescence imaging: hyper-autofluorescence and hypo-autofluorescence [86].

Wu and coworkers developed a conditional GAN, RA-cGAN, using Adam optimization to generate synthetic FAF images from en-face OCT 200 × 200 × 1024 voxels images for automatic geographic atrophy (GA) segmentation [87]. The synthetic FAF and en-face OCT images were then processed in a DCNN to enhance GA segmentation accuracy. By training the RA-cGAN to translate features from en-face OCT to synthetic FAF images while preserving lesion details, the method was shown to improve GA detection using a spatial fuzzy c-means algorithm, leading to superior performance over previous models. Conversely, Su and coworkers generated synthetic FAF images from color fundus images using a Pix2PixHD GAN method, harnessing both imaging modalities to enhance accurate classification and screening of AMD patients [88]. Furthermore, Veturi and colleagues used a GAN-based framework to address the inherent class imbalance in datasets for rare diseases like IRD, erroneously resulting in deep learning models favoring the more prevalent diseases in 512 × 512 resolution images [89]. The authors generated artificial FAF data for nine different classes of IRDs using a StyleGAN2-ADA model. They further proposed the synthetic dataset-trained deep learning-based IRD classifier as a proxy for real data-trained models.

### 7.2. Fluorescein Angiography

Fluorescein angiography (FA) is considered the gold standard for retinal and choroidal circulation assessment. To obtain FA images, a fluorescein dye is injected intravenously before capturing images through a fundus camera equipped with a barrier and an excitation filter. The images are captured in multiple phases: choroidal, arterial, arteriovenous, venous, and recirculation [90]. Depending on the fluorescein patterns, abnormalities can be classified into hyperfluorescent and hypofluorescent images [91]. However, in addition to being invasive, this technique has several limitations. Fluorescein is classified as a category C drug and has teratogenous effects, especially in the first trimester [92]. The risk of allergic reaction from mild skin rash up to anaphylactic shock exists [93]. Therefore, the possibility of generating FA images is highly promising and could potentially leverage the challenges and limitations associated with FA in clinical practice.

Many exploratory investigations have shown the potential of utilizing GANs for translating color fundus photographs to synthetic FA images, notably by using LA-GAN [94], LrGAN and HrGANS [95], Sequence GANs [96], a cGAN with a hierarchal setup [97], Fundus2Angio [98], Attention2AngioGAN [99], and VTGAN [100]. Recently, Kamran and coworkers designed the FA4SANS-GAN architecture for color fundus photography to FA translation for the detection of Spaceflight-Associated Neuro-Ocular Syndrome (SANS) [101]. Their deep learning technology significantly outperformed three other state-of-the-art GAN-based models. With the goal of improving DR screening and providing an alternative when fundus FA imaging is not feasible, Shi et al. used a PixtoPixHD to translate color fundus images into venous and late phase FA images [102]. The same team applied this model on FA and color fundus photography paired images to conduct retinal-vessel segmentation in a finetuned single shot manner using a cross-modality soft AV label pretraining method [103]. Recently, Ge and colleagues explored the translation of ultra-wide field scanning laser ophthalmoscopy (UWF-SLO) images into synthetic ultra-wide field FA (UWF-FA) generated by a novel UWAFA-GAN network. Their methodology captures microvascular pathological changes in superior resolution [104]. Conversely, Wang and colleagues proposed a unified cycleGAN and CNN framework for automated grading of DR. The model differentiated non-proliferative (NPDR) and proliferative DR (PDR) based on the ischemic and leakage indexes and assessed severity with UWF-FA, providing accurate categorization [105]. This method was also shown to yield accurate classification in simulated 7-SF, although not as precise as UWF-FA. Finally, Abdelmotaal and colleagues reported the Pix2Pix GAN network’s capacity to synthesize clinically relevant OCT color-coded macular thickness maps from a modest-sized original FA dataset and the reverse process. The network translation implementation aimed to provide clinically useful alternatives to imaging methods for diabetic macular edema patients (NCT05105620) [106]. Overall, the current advances regarding GAN models for FA image generation are a valuable tool for clinical application. Patients with DR constitute a large proportion of patients undergoing FA testing in clinics. These models can limit the associated risks and psychosocial burden associated with conducting an FA assessment in the clinic.

### 7.3. Indocyanine Green Angiography

Indocyanine green angiography (ICGA) is a widely recognized method for identifying chorioretinal diseases. One key feature of indocyanine is its ability to be visualized through the RPE, lipid exudates, and fluid, making it a better imaging technique to look at the choroid when compared to fluorescein. Indocyanine is also a type of dye with limited leakage from the vessel walls in comparison to fluorescein [107].

Chen and coworkers developed a deep learning algorithm that uses generative adversarial networks for cross-modal data translation and augmentation and assessed its accuracy in classifying AMD [108]. By employing Pix2PixHD GAN, they successfully synthesized high-visual fidelity ICGA early, medium, and late phase images from color fundus images. Their findings indicate that color fundus photography to ICGA translation effectively boosts the accuracy of AMD classification in deep learning studies, dynamically predicts choroidal lesions, and enhances population-based AMD screening. Jiang and colleagues proposed a cGAN-based method to automate linear lesion segmentation in ICGA images [44]. Linear lesions are key indicators of myopic macular degeneration and crucial markers for the progression of high myopia [109]. Additionally, their proposed model outperforms previously studied non-adversarial deep learning networks in lesion segmentation.

### 7.4. Optical Coherence Tomography

OCT is a non-invasive imaging method that allows for the acquisition of a cross-sectional map of the retina within seconds to allow better characterization of lesions and abnormalities within the different layers of the retina [26]. This imaging technique utilizes a concept known as interferometry to provide images with a resolution of 1–15 microns [110]. Time domain, spectral domain, and swept source are three different types of OCT imaging modalities, which differ according to their scanning speed, among other differences [111]. Depending on the location, pattern, and distribution of the hypo- or hyper-reflective changes in the image, ophthalmological diagnoses can be reached.

In recent years, GANs have demonstrated significant potential in enhancing OCT imaging applications across various domains. Image segmentation is studied to pinpoint pathologic regions within identified areas. Wang et al. introduced a CycleGAN-based approach for lesion segmentation in full-width OCT images, achieving an AUC of 96.94% and a DICE coefficient of 0.83239, with a notable image generation time of just 0.039 s, highlighting the method’s efficiency and accuracy in real-time clinical settings [112]. Menten et al. utilized a counterfactual GAN to generate a synthetic longitudinal time series of retinal layers, providing high-resolution and high-fidelity images for studying retinal aging [113]. With the goal of feature extraction and classification, Sun et al. showed that deep learning models trained on a synthetic balanced dataset generated by StyleGAN2-ADA outperformed those trained on unbalanced datasets for retinal condition classification [114].

Additionally, GANs have been extensively studied for synthesizing realistic ophthalmic scans for enhanced data augmentation. In an attempt to expand the training dataset for OCT image classification and drusen body identification, He et al. used a Least Squares GAN (LSGAN) model, concluding that synthetic unlabeled images could improve CNN classifier performance on limited datasets [115]. Kugelman et al. successfully applied cGANs to generate synthetic images, showing improved OCT chorioretinal boundary segmentation [116]. GANs are also used to eliminate noise that can possibly obscure pathologic features in the images, improving on other deep learning technologies that are limited in their ability to produce high-quality denoised images. Wu and coworkers innovated a GAN-trained strategy named ground-truth OCT (tGT-OCT) for speckle-free imaging [117]. In the realm of image enhancement, based on previously researched SiameseGAN, Mehdizadeh and coworkers developed several GAN-based networks to denoise OCT images, preserving visual texture akin to unaltered OCT images while removing noise that could obscure pathological features [118]. Comprehensive evaluations composed of clinician analysis and qualitative and quantitative assessments concluded that the UNet-PatchGAN/WGAN-MSE network outperformed the previous SiameseGAN. Liang et al. contributed to image enhancement by utilizing a CGAN architecture for resolution enhancement in a micro-OCT system [119]. Similarly, Cheong et al. introduced a DeshadowGAN model to remove shadows created by blood vessels in the optic nerve head in OCT images [120]. Halupka and coworkers’ work on speckle reduction using a WGAN further illustrates the improvements in OCT image quality achievable through GANs [121]. Previously, Ren et al. proposed a CycleGAN-based OCT harmonization method that significantly improved image quality in terms of fidelity, sharpness, and contrast, demonstrating superior performance compared to baseline methods [43].

GANs have also been employed for domain transfer and generalization between imaging modalities. Wu et al. demonstrated the harmonization of a GANSeg system across OCT devices without labeled data, achieving segmentation performance comparable to human graders [122]. Similarly, Chen and coworkers introduced a two-stage CycleGAN-based network to standardize retinal OCT images from different devices, significantly boosting segmentation accuracy without the need for manual labeling [123]. Lazaridis and colleagues showed that cyclical GANs could enhance older TD-OCT images to match the signal quality of modern SD-OCT images, improving the reliability of glaucoma progression predictions and improving the statistical power of the UK Glaucoma Treatment Study [124]. Similarly, Romo-Buchelli and colleagues' work on CycleGANs indicated their effectiveness in addressing image variability across different OCT domains, enhancing the generalizability of segmentation models [125].

In an attempt to find a superior method for the diagnosis of macular edema, Tripathi et al. compared multiple GAN models, identifying the top-performing model and super-optimizing said StyleGAN2 using Particle Swarm Optimization (FID 18.84) [126]. The automated model, in pinpointing biomarkers, allows for standardized treatment planning as it forecasts disease status and progression. Another study highlighted the use of RegGAN against six state-of-the-art GAN models for its prognostic accuracy in predicting retina structural changes post-anti-VEGF treatment for diabetic macular edema (DME), assisting clinicians in treatment planning [127]. Post-intervention prediction was also studied by Lee and coworkers using a cGAN network trained on a dataset of baseline OCT-B scans in addition to FA and ICGA images of neovascular AMD [128]. The addition of the beforementioned images to the OCT-B training set resulted in improved post-treatment OCT images of nAMD. Synthetic post-therapeutic OCT imaging for short-term prediction of the same disease was also generated by Liu and coworkers using a Pix2PixHD method [129]. Furthermore, the Pix2PixHD algorithm was shown to accurately predict short-term response to anti-VEGF therapy in patients with retinal vein occlusion by generating synthetic post-therapeutic OCT images [130].

### 7.5. Optical Coherence Tomography-Angiography

OCT-A is also a non-invasive imaging modality utilized to image the microvasculature of the retina and choroid. The technique relies on performing multiple OCT scans one after the other, using the backscattering of light to generate high-quality images. The difference in motion-contrast between each OCT scan is the key point necessary for creating three-dimensional (3D) images. OCT-A images allow visualization of blood flow in the retinal microvasculature, enabling the examination of the choroidal, retinal, and optic nerve blood vessels without requiring dye injections [131]. Other advantages of OCT-A are its reproducibility, as it does not require a skilled operator, and imaging speed since it can produce an image in approximately six seconds [132]. Despite the many advantages of OCTA, this imaging modality has a few limitations. These include its small field of view, the impossibility of accurately displaying leakage, and the possibility of image artifacts from patient movement [133].

Badhon and colleagues leveraged the relationship between OCT and OCT-A imaging to produce translated OCTAs (TR-OCTA) exclusively from OCT data [134]. This 3D-GAN-based framework is proposed to facilitate more accessible characterization of retinal features, which are traditionally limited to the use of costly OCTA devices. Coronado et al. introduced a novel approach to extract detailed retinal perfusion data using fundus images alone by utilizing a cGAN to synthesize en-face 45-degree OCT-A images from local patches of paired fundus and OCT-A images [135]. GANs can also be applied to OCT-angiography to combat the common stripe artifacts and low contrast, limiting the imaging modalities’ diagnostic precision. Cao and colleagues developed a promising enhancement framework constituting a Perceptual Structure Generative Adversarial Network (PS-GAN) to re-enhance synthesized OCTA images previously de-striped by a Stripe Removal Net (SR-NET), preserving vascular integrity [136]. Jiang et al. compared deep learning models, including Pix2PixGAN, for OCTA reconstruction [137].

### 7.6. Electroretinogram

ERG is a non-invasive test used to diagnose various retinal pathologies by measuring the electrical activity of the retina in response to different light stimuli. Multiple parameters are used to interpret an ERG; the waveform components are separated into four components: the a-wave reflects the outer retinal function (corresponding to the early hyperpolarization of the rod and cone photoreceptors), while the b-wave reflects the phototransduction activity corresponding to the positive deflection following the a-wave. Oscillatory potentials reflect the inner synaptic retinal feedback circuits as well as vascular function, and photopic negative response corresponds to the response of the retinal ganglion cells. Those waveforms are then analyzed based on amplitude, implicit time, and latency; then, the b-wave to a-wave ratio is calculated [138]. The full-field ERG reflects the overall functionality of the retina in comparison to a more recent electrophysiologic test called the multi-focal ERG that detects distinct areas of outer retinal damage [139]. One of the main limitations of full-field ERG is the possibility of missing focal retinal disease. This limitation can generally be overcome by using focal ERG or multifocal ERG [138].

In their proof-of-concept study, Kulyabin and colleagues employed cGANs to generate synthetic ERG data from ground truth ERG waveform signals, focusing on increasing the sample size for underrepresented classes to balance and augment datasets [140]. They established that the cGAN architecture could artificially create waveforms of different shapes with characteristics closely mimicking those found in authentic temporal data, including an a-wave, OPs, and b-wave. By increasing the sample size of minority classes, the study showed improvements in classification accuracy, supporting the development of robust models for diverse clinical conditions. Applying machine learning and signal analysis to ERGs may prove particularly beneficial for improving the statistical accuracy of the classification of rare and complex disorders like IRDs and in neurodegenerative and neurodevelopmental conditions that present with variable clinical phenotypes.

### 7.7. Visual Fields

Although standard automated perimetry is the gold standard for assessing visual function, the modality proves challenging with variable results for higher-stage disease or the presence of ophthalmic comorbidities [141]. Although not yet applied to vitreoretinal pathologies, GANs’ accuracy for the modeling of visual field (VF) results has been demonstrated in glaucoma. Hussain and coworkers developed a novel approach to model expected glaucoma progression and VF loss by synthesizing future OCT images using a Pix2PixGAN-based model conditioned on baseline images to predict changes in VF mean deviation 12 months after the initial patient visit. This AI-based approach provides an alternative to glaucoma diagnosis without static perimetry, reducing examination time. Numerous vitreoretinal conditions can lead to VF defects, such as retinal vascular occlusions, retinal detachments, certain retinitis, and macular lesions. Therefore, the possibility of modeling tools to predict the impact of a vitreoretinal disease on VF is highly sought after. For example, projected VF progression in conditions involving retinal vascular occlusions as well as inherited retinal diseases may be helpful in predicting patient outcomes and the impact of medical therapy on visual potential.

## 8. Future Perspectives

With their introduction to ophthalmology, GANs have the potential to change and improve multimodal imaging for vitreoretinal surgeons. With reliable GANs, numerous uses can be extrapolated in clinical practice to generate higher quality images, generate complementing investigations in centers with limited resources, or for teaching purposes. GANs have also been described as great learning tools for trainees. Given the importance of pattern recognition in ophthalmology and especially retinal subspecialties, the use of high-fidelity generative models to generate a vast number of realistic images of various pathologies has the potential to improve the quality and quantity of learning opportunities. This concept could also be expanded not only to fundus imaging but to all associated elements of multimodal imaging and functional testing, including OCT, OCTA, FA, and VF. Progressive GANs are designed to improve image resolution progressively during training. They start by generating low-resolution images and systematically refine details in subsequent training phases. These models have been successfully used to enhance the quality of retinal photographs taken with handheld cameras. Inpainting-specialized GANs are customized to complete images by filling in missing pixels, effectively restoring photos by replacing obscured regions. Furthermore, imaging applications can generate synthetic intermediary slices between two captured slices. This capability could produce thinner slices from OCT scans originally obtained with thicker imaging protocols or generate phases of fluorescein angiography not initially captured. These advancements have the potential to improve diagnostic accuracy and comprehensiveness in medical imaging practices. Although this cannot replace an actual good quality picture, the ability of generative models to optimize imaging modalities allows for mitigation for media opacities affecting their quality (surface disease, cataract, vitreous opacities, etc. GANs have also been shown to be effective for multimodal imaging translation as well as lesion-region segmentation, which could allow for centers with limited resources to make up for outdated devices, low-quality scans, and lack of data points, reduce expenses, and optimize segmentations. Flash ERG and multifocal ERG represent crucial functional imaging modalities for rare and complex retinal pathologies by measuring the electrical response of the retina to light stimulus. However, their use remains limited by the rarity of the disease requiring such investigations, as well as the limited centers and technicians able to perform them. A future application of GAN models would allow for functional imaging from structural imaging translation. This would allow for the generation of ERGs from otherwise readily available imaging modalities, including OCT or fundus photographs.

## 9. Limitations

GANs encounter limitations in requiring substantial data input for the generator to produce convincing images that can deceive the discriminator effectively. Data scarcity poses a significant challenge to implementing GANs in medical imaging, which is critical for modern retinal specialists. Class imbalance also presents a notable issue affecting the accuracy of various neural network architectures. When one class in the training dataset contains significantly more data points than others, the network tends to prioritize learning from the majority class. This can lead to strong performance in majority-class instances but poor performance in minority-class instances. This imbalance is particularly problematic in classification tasks and extends to GANs as well. In non-GAN models, it is advisable to evaluate metrics beyond overall accuracy to ensure that the network’s performance is not adversely affected by class imbalance, thus providing a more comprehensive assessment of model effectiveness. At the heart of machine learning applications is the process of creating neural networks by feeding them data. This concept applies to GANs as well. However, unlike basic DCNNs and CNNs, these networks are highly dependent on their data source. If there is an imbalance between the number of class datapoints, a GAN can become highly functional in that particular class but of limited clinical value for cases outside the presented class. An example of this would be developing a GAN trained on patients for NPDR. Such a GAN would have limited functionality in cases of NPDR. In addition, compared to traditional programming, where distinct rules are written by developers with a transparent understanding of the underlying code, the inner workings of neural networks generated by machine learning approaches are often unclear. In these models, the computational reasoning by the neural network is often a mystery. This is often called the “black box” effect. Attempts have been made to open the black box with heat mapping in DCNNs and feature importance in ANNs. However, minimal successful attempts have been made to open the black box in GANs. Furthermore, the structure of a GAN is that of a competitive network where a discriminator function and a generator function compete to outperform one another. This typically results in improving the performance of both networks. However, if the generator model begins to outperform the discriminator, then the discriminator may provide poor feedback to the generator, decreasing its performance. Furthermore, if a generator learns to produce images from one class very well but fails with other classes, then it will consistently obtain positive feedback from the discriminator for producing one image class and negative feedback for producing images for other classes. As a result, the generative model will only produce images for one image class. For example, if a GAN designed to generate FAs is given a dataset of fundus photos for both NPDR and PDR consistently fails at producing appropriate FAs for PDR and succeeds at generating images for NPDR, then the generator model may choose not to produce PDR FAs even when appropriate. Fundamentally, the competitive design of these networks allows for improving performance through training cycles; however, such a structure can still result in error.

Importantly, GANs are inherently challenging to evaluate. Unlike classification problems where specificity, sensitivity, and total accuracy can be measured and compared easily, the output of GANs is an image judged based on realism. Such a term is poorly defined, and many have struggled to develop a standard agreed-upon metric for evaluating GANs. With the advent of AI, we have seen means of determining patients’ sex, age, and blood pressure with a simple picture of their respective fundus. As such, this could, in turn, limit the usability of numerous scans as the data would then need to be encrypted or protected. With the increased interest in AI’s implementation in medical imaging, there are rising concerns for patient data privacy protection.

## 10. Tackling the Challenges Associated with Generative Adversarial Networks

### 10.1. Vanishing Gradients

Vanishing gradients are observed during model training when the gradients of the loss function used in gradient descent become very small, resulting in minimal updates of weights in the neural network during back propagation [53]. This slows the training process and can result in the early termination of generative network training [53]. In GANs, vanishing gradients can be observed if the discriminatory model is too effective: the generative model will always receive negative feedback, and a functional generative network will not be created as the network's training will terminate early [53]. A simple approach to prevent vanishing gradients in GANs includes the use of Wasserstein loss and modified minmax loss [142,143].

### 10.2. Failure to Converge

As described earlier, if the functionality of the generator model surpasses that of the discriminator model, then the generator model may receive inappropriate feedback. In order to counter this, a multitude of techniques have been employed [69]. Similar to vanishing gradients, the use of Wasserstein loss and modified minmax loss can be used to prevent failure to converge [142,143]. If failure to converge happens with Wasserstein loss or other models, one could include both the generator model outputs and known false negatives in discriminator training to provide the discriminator with a consistent flow of negative results [142,143]. Furthermore, regularization schemes can be used: they penalize the weights from the discriminator function as the functionality of the generator improves over the discriminator [144].

### 10.3. Mode Collapse

An ideal GAN is expected to produce a variety of images, and not only a few that are non-diversified [58,145]. Failure to achieve that capability can occur due to model collapse [58,145]. Model collapse can occur when the generative model, trying to outperform the discriminatory model, produces an output that is very close to ground truth [58,145]. If the discriminator is unable to discriminate between the fake and real image and the generator continues to present this same (or too similar) image over and over, only a few non-diverse images will be produced by the GAN [58,145]. For example, if a GAN developed to perform data augmentation by producing multiple variants of a rare form of retinal malignant tumor produces, after complete training, only a set of ten identical new images of the rare retinal malignancy, then we can assume that the model collapse happened to the model. To avoid undiversified output from a generative model, a large, diverse training and testing set should be employed [62,64,146,147]. Furthermore, the two following methods can be used [62,64,146,147]. First, using Wasserstein loss allows the discriminator to reject discriminators’ solutions on which the generators want overfit [62,64,146,147]. This rejection forces the generator to propose new outputs [62,64,146,147]. Second, some GANs, called unrolled GANs, employ more than a single discriminator network, which prevents the generative model from overfitting to a single discriminator [62,64,146,147].

### 10.4. Class Imbalance

Class imbalance occurs when a neural network is not exposed enough to one or several subclasses of the dataset due to insufficient variety in the input provided and learns to be biased towards the dominant class provided [69]. The model performs well when dealing with cases from the majority class and poorly when dealing with cases from the minority class [69]. Class imbalance significantly reduces the accuracy of multiple neural network architectures and can be a challenge in the medical field, where some diseases and conditions are rare [69]. In the case of a GAN built for predicting FA results, if the training set is overly sampled with cases of NPDR, then the output results of PDR would likely be few and of poor quality [69]. To avoid this, data should be collected as equally as possible for each of the target output classes. In non-GAN models, metrics other than total accuracy should be evaluated to ensure a network is not hampered by class imbalance [69]. A very interesting and important fact is that a major utility of GAN is data augmentation, which is to artificially create new data from available data with the objective of training AI models [69]. In other words, in the example of FA, if a dataset has few cases of PDR, a first GAN can perform data augmentation and generate synthetic images of PDR, which will serve a second GAN that will potentially predict FA results more accurately, minimizing class imbalance [69].

### 10.5. Unintended Bias

Unintended bias refers to learning unintentionally acquired by a neural network that negatively impacts its performance [148,149,150]. Unintended bias can occur in multiple steps of training, including during data input and data evaluation [148,149,150]. Bias is very similar to class imbalance. Data input has either underrepresented parts or has intrinsic biases if there is a discrepancy between training input and actual input for which the data are evaluated [148,149,150]. For example, a model trained for a specific retinal malignancy in today’s population may perform differently if used in 40 years in a population where the same specific retinal malignancy is manifesting itself differently due to a change in risk factors and exposures. Bias may occur when sample image datasets are small and without appropriate spatial alignment, in which case GANs may struggle to learn spatial alignment accurately. For this reason, spatial distortions are often incorporated into GAN outputs [148,149,150].

### 10.6. Hyperparameter Sensitivity

In machine learning, parameters refer to internal variables of the model that fluctuate and are determined through a training session [151]. In contrast, hyperparameters refer to variables that determine the neural network’s structure and need to be defined before training [151]. Hyperparameters include the number of nodes in the hidden layers, the learning rate, the number of iterations of training, etc. Some GANs can be sensitive, and small changes in hyperparameters can have significant implications for the model’s performance [151]. A GAN developer should be aware that slight variations in hyperparameters could drastically affect performance [151]. Thus, determining hyperparameters by trial and error is not always a good approach [151].

### 10.7. Data Dependence

Data dependence is the reliance of a neural network's performance on the underlying data [152,153]. As machine learning is based on developing an algorithm in response to data, models can be highly susceptible to poor data quality [152,153]. This is often more so in more complicated models. GANs are typically more data-dependent than simpler models such as DCNNs, CNN, and ANNs [152,153]. For example, an ANN may be able to function with relatively high accuracy with a 10% mislabeling rate in the training data [154,155]. However, such an error rate in a GAN training set may hinder performance significantly [154,155].

### 10.8. Summary

Vanishing gradients, failure to converge, mode collapse, class imbalance, unintended bias, hyperparameter sensitivity, and data dependence are key challenges in training GANs. Strategies such as using Wasserstein loss, unrolled GANs, diverse training data, and regularization can mitigate these issues. Additionally, addressing biases, hyperparameter tuning, and ensuring high-quality data are critical for optimizing GAN performance and avoiding unintended consequences. While physicians should be aware of these challenges, their resolution ideally requires collaboration with experts in machine learning and neural network optimization to ensure robust and reliable outcomes in medical AI applications.

## 11. Conclusions

GANs have significant potential in the field of medicine, mainly fields like medical and surgical retina, where imaging modalities play a key role in the diagnosis and prognosis of disease. The structure of GANs differs from that of typical machine learning programs used in the field, and, therefore, the flaws associated with these networks need to be acknowledged. There is no one-size-fits-all approach to determining the structures of GANs; therefore, the structure of a GAN implemented in a project should be determined on a case-by-case basis. The primary variables to consider in developing a GAN can include the data structure, the CNN or DCNN structure, the loss function, and the evaluative metrics. There are a multitude of GAN architectures that can be used in conjunction with each other to build a custom GAN for a specific application. Still, the most significant challenges associated with GANs are data collection and quality. Without an appropriate training dataset, a GAN is unlikely to succeed. Therefore, before a GAN is developed, researchers should be aware of their own data limitations. Furthermore, the differing structure of GANs results in differing challenges and limitations compared to other machine learning models. Awareness of these limitations is essential to avoid these limitations. One key limitation is in the evaluation of these networks as there has yet to be a gold standard metric to judge the quality and realism of GANs outputs. Despite this, GANs have significant promise in medical imaging, and it is likely that such technologies will be implemented in the field of ophthalmology in the near future.

## Figures and Tables

**Table 1:** Basic terminology of generative adversarial networks and other artificial intelligence-based algorithms.

Term	Explanation	References
AI ^a^ model	A program that has been trained to perform a task without further human intervention. For example, this task can be as simple as telling if the number provided (called input) is greater or less than a certain integer (i.e., 5).	[44]
Neural network/artificial neural networks	Type of AI model that uses several layers of nodes (artificial neurons) to perform a task. The first node layer is the input layer, followed by one or several layers of “hidden nodes”, and the last layer is the output. Nodes in one layer are related to nodes in previous layers by weights and biases that summarize the input signal and an activation function that determines whether that signal should be transmitted.	[6,7]
Convolutional Neural Network (CNN) ^a^	A CNN ^a^ is a type of neural network adapted for image-type inputs and data that is in the form of a grid. Their major particularity is that they apply a matrix (i.e., a 3 × 3 grid matrix) and convolutional operations to input data.	[4,5]
Deep convolutional neural networks	A type of neural network similar to CNNs, but with a larger architecture allowing the model to perform more complex tasks. This gain in terms of accuracy and complexity costs more computer power.	[3]
Discriminatory models	AI models that perform classifications and output labels. These models help with discriminatory tasks, such as, but not limited to, distinguishing healthy versus pathological conditions, benign versus malignant lesions, disease A versus disease B, etc.	[45]
Generative models	AI models that learn patterns from a dataset and output new but similar data. In imaging generative models, the output is often a new image rather than a predefined label.	[46,47]
Generator	The first part of a GAN that uses an internal distribution of imaging data is to create a candidate image close to a concrete real-world counterpart. The objective of training is to maximize the generator’s performance.	[47]
Discriminator	The second part of a GAN tries to distinguish between real and fake data provided by the generator. The objective of the training is to have a discriminator that is unable to distinguish between real versus fake data provided by the generator while maximizing the discriminator’s performance.	[47]
Backpropagation	A mathematical update of weights from the last to the first layer of the AI model based on the loss function. The partial derivatives of the loss function to each weight are used in the weight update. This mathematic type of optimization is called gradient descent. The objective of backpropagation and gradient descent is to maximize the models’ accuracy by minimizing the loss function.	[48]
Loss function	A function that evaluates the algorithm’s performance when comparing its output to the answers (ground truth). Incorrect answers provide a high loss value. The objective of training is to minimize the loss function.	[49]
Epoch	Corresponds to one algorithm training session with a complete pass of the training dataset.	[50]
Overfitting	Undesirable behavior of an algorithm that fails to generalize from input data by being too specific and fitting too closely. The algorithm is thus unable to correctly perform accurate prediction.	[51]
Convergence	Corresponds to the point where the loss function is successfully minimized, and training parameters reach a stable state. The algorithm is thus able to accurately perform predictions with the obtained parameters.	[52]

^a^ Abbreviations: AI, artificial intelligence; CNN, convolutional neural networks; GAN, generative adversarial network.
